# Major Reduction in Anti-Malarial Drug Consumption in Senegal after Nation-Wide Introduction of Malaria Rapid Diagnostic Tests

**DOI:** 10.1371/journal.pone.0018419

**Published:** 2011-04-06

**Authors:** Sylla Thiam, Moussa Thior, Babacar Faye, Médoune Ndiop, Mamadou Lamine Diouf, Mame Birame Diouf, Ibrahima Diallo, Fatou Ba Fall, Jean Louis Ndiaye, Audrey Albertini, Evan Lee, Pernille Jorgensen, Oumar Gaye, David Bell

**Affiliations:** 1 Programme National de lutte contre le Paludisme, Ministère de la Santé, Dakar Fann, Senegal; 2 Faculté de Médecine, Université Cheikh Anta Diop de Dakar, Fann Dakar, Sénégal; 3 Foundation for Innovative New Diagnostics (FIND), Geneva, Switzerland; 4 Global Malaria Programme, World Health Organization, Geneva, Switzerland; Lile 2 University, France

## Abstract

**Background:**

While WHO recently recommended universal parasitological confirmation of suspected malaria prior to treatment, debate has continued as to whether wide-scale use of rapid diagnostic tests (RDTs) can achieve this goal. Adherence of health service personnel to RDT results has been poor in some settings, with little impact on anti-malarial drug consumption. The Senegal national malaria control programme introduced universal parasite-based diagnosis using malaria RDTs from late 2007 in all public health facilities. This paper assesses the impact of this programme on anti-malarial drug consumption and disease reporting.

**Methods and Findings:**

Nationally-collated programme data from 2007 to 2009 including malaria diagnostic outcomes, prescription of artemisinin-based combination therapy (ACT) and consumption of RDTs in public health facilities, were reviewed and compared. Against a marked seasonal variation in all-cause out-patient visits, non-malarial fever and confirmed malaria, parasite-based diagnosis increased nationally from 3.9% of reported malaria-like febrile illness to 86.0% over a 3 year period. The prescription of ACT dropped throughout this period from 72.9% of malaria-like febrile illness to 31.5%, reaching close equivalence to confirmed malaria (29.9% of 584873 suspect fever cases). An estimated 516576 courses of inappropriate ACT prescription were averted.

**Conclusions:**

The data indicate high adherence of anti-malarial prescribing practice to RDT results after an initial run-in period. The large reduction in ACT consumption enabled by the move from symptom-based to parasite-based diagnosis demonstrates that effective roll-out and use of malaria RDTs is achievable on a national scale through well planned and structured implementation. While more detailed information on management of parasite-negative cases is required at point of care level to assess overall cost-benefits to the health sector, considerable cost-savings were achieved in ACT procurement. Programmes need to be allowed flexibility in management of these funds to address increases in other programmatic costs that may accrue from improved diagnosis of febrile disease.

## Introduction

The World Health Organization recently strengthened its recommendation for parasite-based diagnosis of malaria, extending it to all cases of suspected malaria prior to treatment with anti-malarial medicines [1] Accurate diagnosis enables targeting of anti-malarial drugs to those who will benefit, early identification of non-malarial fever requiring alternative management, and accurate and complete surveillance for confirmed malaria cases. Reducing drug wastage, in addition to saving money and conserving stocks of artemisinin-based combination therapies (ACT), may prolong the usefulness of ACTs globally by reducing pressure towards resistance. Clinical (symptom-based) diagnosis of malaria has a very poor specificity [2], [3], [4], and microscopy is predominantly limited to larger health facilities where the quality of the result can be assured [5]. Provision of universal access to parasite-based diagnosis for populations at risk of malaria will therefore depend on the wide use of malaria rapid diagnostic tests (RDTs); point-of care tests first introduced in 1993 (with the *ParaSight-F* test) and with a proliferation of products now coming into wide use [6], [7], [8], [9].

Rapid point-of-care tests are routinely used for several diseases including HIV and syphilis, replacing centralized laboratory testing, as the requirement for a positive diagnostic result has long been accepted as a basis for treatment. , However, due to the historical anomaly of wide use of poorly-targeted anti-malarial treatment based on symptoms, particularly in sub-Saharan Africa, the introduction of malaria point-of-care tests will lead to a restriction in availability of treatment, rather than a widening of access as is intended through the introduction of HIV and syphilis test. Health care providers (and the communities they serve) must now not only re-learn malaria diagnosis, but develop and implement new management strategies for the majority of febrile patients whose malaria test results will be negative. This poses a dilemma for resource-limited health services, as diagnostics for non-malarial febrile illness are often unavailable and management strategies limited. Further, difficulties in ensuring malaria RDT quality promoted doubt as to whether the RDT was sufficiently accurate as a basis for with-holding a potentially life-saving anti-malarial drug. The establishment of a product testing programme [9], methods for laboratory-based RDT lot-testing [10], and evidence of safe withholding of treatment in the field [11], [12], have addressed many of these concerns.

Although modeling suggests that malaria RDTs will be cost-effective, and the potential public health advantages of enabling early appropriate management for other causes of fever are clear, these outcomes depend heavily on adherence to test results by providers and patients and in access to effective management of non-malarial fever [13], [14]. Reported adherence to results has varied in reported studies; some studies questioned whether RDT use on a large scale can have a significant impact on the management of febrile disease [15], [16]. Successful implementation will depend on a number of factors including good training of health workers and modification of long-standing community and clinician attitudes to the causes and management of febrile disease [14], [17], [18], [19].

While RDTs have been used on a large scale for several years in some countries with anecdotal and unpublished health system data suggesting an impact on anti-malarial therapy, these outcomes are poorly documented and opinion on the advisability of wide-scale use has remained divided [20], [21]. Here we report the impact of the comprehensive national roll-out of RDTs in Senegal, and its impact on management of malaria in that country.

### The Senegal Programme

Malaria is endemic throughout Senegal. *Plasmodium falciparum* accounts for virtually all reported cases [22]. Approximately 75% of patients access public health facilities for management of fever [23], the vast majority doing so through peripheral health huts (“cases de santé”) rather than hospitals or clinics with established laboratory capacity. Artemisinin-based combination therapy (ACT) was introduced as first-line therapy in 2006 (artesunate-amodiaquine; AS-AQ). Until 2007 malaria diagnosis was predominantly based on clinical assessment, with microscopy-based diagnosis limited to hospitals. Of 1555310 reported fever cases at public health facilities in 2006, only 3.1% (48275) were confirmed to be malaria by microscopy (Data records, Senegal National Malaria Control Programme – NMCP).

**Figure 1:**
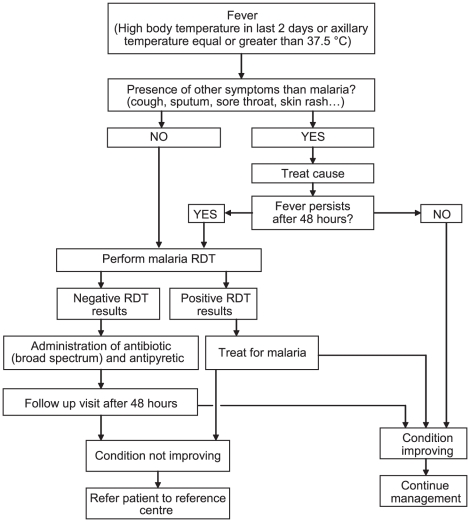
Malaria Case Management Algorithm of the Senegal NMCP, introduced from July 2007.

From September 2007, the use of malaria RDTs was incorporated by the NMCP into a revised national policy for management of febrile illness. ACT was to be restricted to confirmed malaria cases as RDTs became accessible. Implementation involved all public sector health facilities beyond hospital level; 78 health centres, 1018 health posts and subsequently all 1640 health huts. This policy calls for the use of RDTs for uncomplicated cases of fever without presence of other symptoms suggestive of non-malarial aetiology. RDT-positive patients are to be prescribed an antimalarial, and RDT-negative patients may be prescribed broad spectrum antibiotics (trimethoprim-sulfamethoxazole or amoxycillin) and antipyretics (Figure 1). Drug costs to the patient were US$0.60 for an adult course and US$0.30 for a paediatric course of antimalarials during the period covered by this report (although antimalarials are now free of charge since May 2010). Costs of antibiotics and antipyretics were US$0.60 and US$0.20, respectively. Follow-up should occur after 48 hours to confirm improvement or a need for referral (Figure 1). Cases ineligible for an RDT (symptoms or signs of other aetiology) are to be managed appropriately but tested with an RDT if still febrile after 48 hours. Microscopy is reserved for complicated cases only (e.g. severe cases or suspected treatment failures, usually after an initial RDT-based diagnosis) [24]. RDT-based diagnosis is provided free of charge to patients in public health facilities.

The introduction of malaria RDTs in 2007 was preceded by scale-up of long-lasting bed-nets and indoor residual spraying (IRS) (Table 1). The RDTs (*Paracheck Device*, Orchid Biomedical Systems, India) were piloted on a limited scale by the national, programme and the University of Cheikh Anta Diop (Laboratory of Parasitology) in Dakar, during which training materials were developed based on generic job-aids and training manuals available from WHO [19], [25]. This RDT met performance criteria for WHO procurement recommendations in the WHO product testing laboratory evaluation, and has demonstrated good accuracy in the field in Senegal [9], [26]. Health worker training in case management and the Senegal national treatment guidelines was conducted by district and regional management teams developed by the NMCP and the University.

**Table 1:** Key dates in introduction of anti-malaria interventions in Senegal.

Intervention	Year of introduction
Indoor residual spraying: primary vector control intervention	1998
Insecticide-treated bednets (more recently long-lasting nets)	2002
Intermittent prophylactic Therapy for pregnancy (IPTp)	2004
Artemisinin-based combination therapy	2006
Rapid diagnostic tests (RDTs)	2007 (Sept)
RDT country ‘full coverage’ (roll-out to health posts, then health huts)	2008 (Late)

During the introduction of RDTs, the definition of ‘malaria-like fever’ was formalized to improve targeting of RDTs (Figure 1) and a vetting process put in place to verify accuracy of reporting and collation of health information system data related to malaria, including consumption data on ACT and RDTs. All malaria RDTs are quality controlled after arrival in Senegal through lot-testing at the Parasitology laboratory of the University of Anta Cheikh Diop (Dakar) prior to dispersal to the field, based on the protocol of the WHO Methods Manual [27]. Microscopy preparation follows the Earl-Perez method described in the same Manual [10].

## Methods

The evaluation is based on routinely-collected and collated programme data, retrieved by the national malaria control programme (Programme National de lutte contre le Paludisme) and the Faculté de Médecine, Université Cheikh Anta Diop de Dakar in 2010. The national malaria surveillance system is based on passive case detection. Data on suspected and laboratory confirmed malaria cases, as well as data on consumption of anti-malarial treatment are notified/reported monthly to the district malaria programme by all levels of the public health system (hospitals, health centres, health posts and health huts) using a standard reporting form. At district level, the reported data is entered into a database (Epi Info Version 6) and send to NMCP on a monthly basis. The NMCP stipulates that the quality of malaria surveillance data is assessed regularly at two levels of the system: Firstly, supervisors from the district malaria programme perform quarterly or bi-annual visits to health care facilities within their district area to cross-check patient records at the facility with data reported to the district from the facility; secondly, aggregated data received from each district are reviewed by NMCP personnel, regional and district malaria programme staff at quarterly meetings.

For the purpose of this programme evaluation, the following monthly data were extracted from the national malaria surveillance database for the period January 2007 to December 2009: i) number of malaria-like febrile disease (‘suspected malaria’) cases, defined as persons with fever (clinically determined or axillary measured temperature ≥37.5°C; ii) number of persons tested for malaria by microscopy or RDT; iii) number of malaria cases confirmed by microscopy or RDT; iv) number of persons treated with ACTs; and, v) total number of all-cause consultations.

We calculated the proportion of persons tested by RDT or microscopy among all persons with suspected malaria and the proportion of persons treated with ACT among all suspected malaria cases in order to assess the impact of universal parasite-based diagnosis using malaria RDTs on anti-malarial drug consumption over a two-year period. The number of ACT courses averted were estimated by subtracting the actual ACT consumption in September 2007–December 2009 from the predicted consumption derived from the proportion of malaria-like fever cases treated with ACTs in January–August 2007 (prior to RDT introduction).

Suspected malaria is reported here as recorded by the programme, irrespective of tightening of the definition from late 2007 that was likely associated with re-training on RDT introduction. The ‘confirmed malaria rate’ is derived from reported confirmed malaria (microscopy-based) up to August 2007, and from the RDT positivity rate after this time, as most microscopy performed after August 2007 involved referred cases previously screened with RDTs. This avoids double-reporting of such cases but will cause a small artefactual decline in late 2007, and possibly a minor underestimation of case numbers thereafter, but as microscopy was performed on only a small subset of patients (5.2% of tested patients through 2009), any underestimation will be small.

## Results

**Figure 2:**
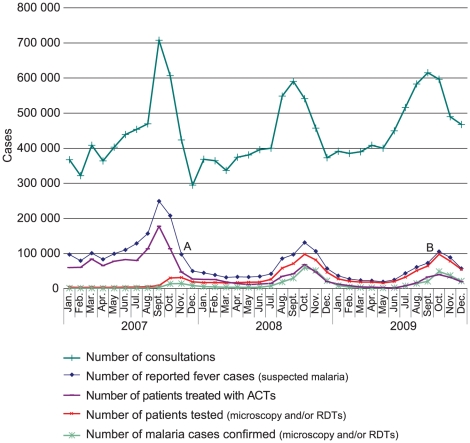
Evolution of parasite based diagnosis of malaria in Senegal public health services 2007–2009. A: Introduction of new case definition for suspected malaria. B: Partial stock-out of ACT due to late replacement of expired stock in some clinics.

In Senegal from 2007 to 2009, 2784532 suspected malaria cases were reported at public health facilities. Case rates followed a clear seasonal trend with an increase in suspected malaria from August to December, accompanied by an increase in both parasite-negative malaria-like febrile disease and in total consultations unrelated to malaria-like fever over the same (wet season) months (Figure 2). As the programme moved from predominantly symptom-based treatment in 2007 to parasite-based treatment in 2009, the frequency of ACT use declined from 67.7% of the malaria-like fever cases (suspected malaria) in 2007 to 31.5% in 2009. Over the same period, the rate of diagnostic testing of malaria-like fever rose from 4.0% and 6.2% by microscopy and RDT respectively in 2007 (RDTs having been introduced after August), to 5.2% and 86.0% respectively in 2009, rising to 96% in December of that year (Figure 2).

**Figure 3:**
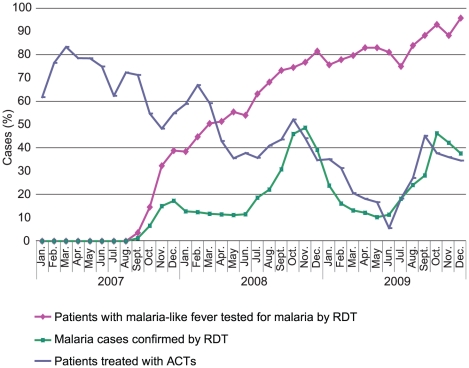
Management of suspected malaria in Senegal public health services, 2007–2009.

Throughout 2009, ACT consumption closely followed the confirmed malaria rate (test-positive rate), apart from a marked trough in ACT consumption in June corresponding with a temporary stock-out in many clinics (Figure 3). During 2009, 174890 RDT-positive malaria cases were recorded and 184170 doses of ACT dispensed.

Table 2 summarizes diagnostic results and the corresponding reduction in ACT courses dispensed and probable courses averted from 2007 to 2009. Taking the period of 2007 prior to RDT introduction (January to August) as a baseline, during which 72.9% of suspected malaria cases were treated with ACTs, the estimated unnecessary ACT courses averted by the programme rose to 249184 in 2009 (Table 2). An estimated 516576 courses of ACT were averted over the entire 3 year period.

**Table 2:** Malaria case management in Senegal, January 2007 to December 2009.

		Reported suspected malaria cases	Suspected malaria cases tested^a^ (%)	Suspected malaria cases confirmed^a^ (%)	Cases of suspected malaria treated with ACTs^b^ (%)	Estimated ACT courses averted^c^
Before introduction of RDTs	Jan–Aug 2007	857179	33263 (3.9)	12468 (1.5)	624601 (72.9)	N/A
After introduction of RDT s	Sept–Dec 2007	605066	90313 (14.9)	40178 (6.6)	365740 (60.5)	75353
	2008	737414	487188 (66.1)	217096 (29.4)	338335 (45.9)	199239
	2009	584873	502739 (86.0)	174890 (29.9)	184170 (31.5)	241984

a Tested by microscopy up to August 2007, and RDT only from September 2007. After August 2007, only RDTs became the first-line diagnostic test and microscopy was confined to referral centres and results were likely to involve re-testing of a case. In 2009, 30414 cases were tested by microscopy.

b Artemisinin-based combination therapy.

c Based on treatment rate of malaria-like febrile disease (suspected malaria) in 2007 prior to rapid diagnostic test introduction.

## Discussion

These data demonstrate a significant decrease in reported malaria cases on a national scale after implementation of parasite-based diagnosis for malaria, with a corresponding reduction in ACT consumption. Of similar importance, the introduction of the strategy of universal diagnosis has provided a high degree of certainty on malaria incidence throughout Senegal. This certainty is enabling the national programme to accurately predict anti-malarial drug requirements and creating the ability to concentrate resources on areas of higher malaria burden and need, and to assess the impact of future changes in intervention rates with insecticide treated bednets and indoor residual spraying.

Prior to RDT introduction, microscopy was used in only a small proportion of suspected malaria cases. Although the definition of reported ‘malaria’ varied, the vast majority of reported cases were based on non-specific symptoms and were treated with ACTs. These figures for assumed malaria were the basis on which the programme had forecast ACT consumption and base procurement. With the introduction of RDTs, subsequent utilization of ACTs was well below the predicted rate, and large stocks of ACTs expired in mid-2009 (delays in replacement resulting in the reported stock-out in some clinics, which accounts for the drop in ACT consumption below the confirmed malaria rate at that time). While the reported malaria-like fever rate remained high despite a tightening of the clinical definition, the near-universal use of RDTs in these cases has provided the national malaria control programme with a solid basis for predicting drug consumption. Senegal can now procure an appropriate quantity of ACTs - a small proportion of the volume previously required. In 2009, the Global Fund retained 1201764 Euros (∼US$1.57) in unused funds allocated for ACT procurement within the grant previously agreed for the Senegal programme.

The reduction of ACT use to near-equivalence with the confirmed malaria rate took some time. Obviously, the gradual introduction of RDTs across the public health sector, necessitated by the requirement to train health workers in RDT use, partly explains this [19]. Eighteen months elapsed before ACT consumption closely tracked the RDT-positive rate, by which time RDT consumption had risen above 80% of the reported malaria-like fever rate. Experience of poor compliance elsewhere suggests that non-adherence to RDT results may also have been responsible for the lag [15], [16]. However, by mid-2008, high adherence with diagnostic results was achieved and continues to be sustained on a national scale.

The high adherence rate to RDT results is likely due to a combination of factors. The Senegal programme, somewhat unusually, charged patients diagnosed with malaria for first-line anti-malarial drugs. While the cost to the consumer was small, this is likely to have contributed to adherence to diagnostic results (non-treatment of RDT-negative cases). The cost of antibiotics was higher than anti-malarial drugs, but only certain RDT-negative patients were prescribed these (NMCP). Other major contributors to adherence are likely to include: (1) a history of prioritizing malaria diagnosis at a central and academic level in Senegal which may have eased acceptance among planners and senior physicians, despite the limited reach of microscopy [28]; (2) strong Ministry of Health support for the programme, backed by policy change and combined with a strong public sector (which is the first point of access for most febrile patients) [23]; (3) tested training materials and job-aids specifically adapted by the NMCP for use at the district and community level [19]; a comprehensive supervisory programme that maintains contact with peripheral health workers; (4) an aggressive roll-out schedule sufficient to achieve near-blanket national public-sector coverage in a relatively short time, with RDT use thereby becoming the norm rather than confined to certain clinics or regions; and (5) a quality assurance system based on lot-testing of RDTs, capable of demonstrating that the particular product used was working prior to deployment and thereby at least partly allaying fears of false-negative results. The University of Cheikh Anta Diop also actively collated and disseminated data to community organizations and NGOs to build public awareness of the change in national malaria policy and guidelines, and engaged key opinion leaders to advocate for RDT use.

While considerable cost savings have been achieved by the Senegal NMCP by reducing unnecessary ACT procurement, overall financial costs to the health service are unclear. Senegal's procurement costs per course of ACT (AS-AQ) averaged US$1.12 per course. As patients paid $0.60 and US$0.30 for adult and paediatric courses of treatment respectively, the overall cost to the programme, ignoring logistical costs, is approximately US$0.70. Cost savings through reductions in ACT use will have been offset by costs in RDTs (similar to the adult ACT course cost-to-programme). Costs of antibiotics that can be provided to RDT-negative patients are higher; full courses of amoxicillin and cotrimoxazole cost the programme about US$2.00 and paracetamol US$1.00, while US$0.60 and US$0.20 is recouped from patients. Figures on antibiotic dispensing prior to and after RDT use were not available, but anecdotal evidence indicates that many RDT-negative patients, probably appropriately, do not receive them. While a limited evaluation in central Senegal indicated that overall cost savings are likely to accrue [26], a full cost-benefit analysis would need to take into account the likely benefit of earlier appropriate management of non-malarial febrile illness where this occurs, and the wider benefits of improved targeting of health interventions enabled through the availability of more accurate incidence data.

Modelling elsewhere suggests that an overall cost-benefit may be expected when RDTs replace presumptive therapy, but these rely on ACT costs higher than those of Senegal, and improved management of non-malarial febrile illness is important to achieving these benefits [13], [29], [30]. Other modelling and field experience also suggests that overall cost implicaitons will be relatively neutral [14], [31], Through partial recoupment of antibiotic costs from the consumer, the Senegal programme will have limited the impact on less-well funded areas of the health system that fund antibiotic prescription. However, funds saved on ACT conservation could also be spent in future on support for non-malarial fever management if more flexibility was allowed by external agencies in the use of allocated funds.

Irrespective of financial loss or gain, the public health imperative of not mis-leading patients or their carers into reliance on an inappropriate three-day course of anti-malarial medication, ineffective for their illness, is clear. Resultant delays in achieving a correct diagnosis and appropriate management may increase mortality from other potentially fatal or debilitating infections. Mortality due to non-malarial febrile disease is twice that of malaria globally, with malaria-endemic countries accounting for a large proportion of this burden [32]. Thus, a basic public health good is at issue, not just a possible benefit in terms of financial cost.

As malaria declines through much of sub-Saharan Africa [22], the need to differentiate malaria from non-malarial fever becomes more pressing. It is too early to confirm from the Senegal data whether an overall decline in malaria is occurring, or whether the apparent decline seen in Figure 2 reflects only better discrimination of malarial from non-malarial febrile illness, initially through a tightening of clinical criteria and now by demonstration of parasitaemia. Interestingly, the introduction of RDTs imposed an additional burden on health workers on diagnosing ‘malaria-like fever’; they now must perform a finger-prick and RDT prior to further management and this may result in at least a sub-conscious reduction in readiness to assign this diagnosis to febrile patients. A decline in reported annual malaria deaths during this period from 1935 to 722 indicates that any increased reluctance to test for malaria was not resulting in poorer outcomes [22]. Alternatively, the downward trend in both non-malarial fever and in confirmed malaria could be real, due to environmental changes over this period affecting multiple pathogens or, speculatively, due to reduced rates of malaria parasite carriage resulting in improved health and resistance to other pathogens. RDT consumption remains below the reported malaria-like fever rate, largely accounted for by the incremental roll-out of RDTs to clinics, this gap reducing toward the end of 2009 as RDT use reached 96% of malaria-like fever cases (Figure 2). As ACT consumption ends 2009 marginally above the RDT-positive rate, these undiagnosed cases are likely to account for much of the remaining over-prescribing of ACT at this time.

The experience in Senegal demonstrates that parasite-based diagnosis reliant on the use of malaria RDTs can be successfully introduced on a national scale and dramatically reduce ACT consumption. In the presence of a strong public health sector, and possibly influenced by some financial incentive to the consumer, RDTs have been used to transform the accuracy of malaria case reporting and open new possibilities for addressing non-malarial febrile illness and manage other causes of morbidity and mortality. The ability to track the identify cases and track the impact of anti-malarial interventions in this way through the widespread use of parasite-based diagnosis will enable malaria elimination to be seriously contemplated., but more flexibility is required in management of funds saved from ACT procurement that will be required to address increased programmatic costs in other areas.
